# Prediabetes prevalence and awareness by race, ethnicity, and educational attainment among U.S. adults

**DOI:** 10.3389/fpubh.2023.1277657

**Published:** 2023-12-18

**Authors:** Taynara Formagini, Joanna Veazey Brooks, Andrew Roberts, Kai McKeever Bullard, Yan Zhang, Ryan Saelee, Matthew James O'Brien

**Affiliations:** ^1^Department of Family Medicine, University of California San Diego, San Diego, CA, United States; ^2^Department of Population Health, University of Kansas School of Medicine, Kansas City, KS, United States; ^3^University of Kansas Cancer Center, Kansas City, KS, United States; ^4^Division of Palliative Medicine, University of Kansas School of Medicine, Kansas City, KS, United States; ^5^Aetion Inc., New York, NY, United States; ^6^Division of Diabetes Translation, National Center for Chronic Disease Prevention and Health Promotion, CDC, Atlanta, GA, United States; ^7^Department of Medicine, Division of General Internal Medicine and Geriatrics, Northwestern University Feinberg School of Medicine, Chicago, IL, United States

**Keywords:** prediabetes, prediabetes awareness, diabetes-related disparities, race and ethnicity, educational attainment

## Abstract

**Introduction:**

Racial and ethnic minority groups and individuals with limited educational attainment experience a disproportionate burden of diabetes. Prediabetes represents a high-risk state for developing type 2 diabetes, but most adults with prediabetes are unaware of having the condition. Uncovering whether racial, ethnic, or educational disparities also occur in the prediabetes stage could help inform strategies to support health equity in preventing type 2 diabetes and its complications. We examined the prevalence of prediabetes and prediabetes awareness, with corresponding prevalence ratios according to race, ethnicity, and educational attainment.

**Methods:**

This study was a pooled cross-sectional analysis of the National Health and Nutrition Examination Survey data from 2011 to March 2020. The final sample comprised 10,262 U.S. adults who self-reported being Asian, Black, Hispanic, or White. Prediabetes was defined using hemoglobin A1c and fasting plasma glucose values. Those with prediabetes were classified as “aware” or “unaware” based on survey responses. We calculated prevalence ratios (PR) to assess the relationship between race, ethnicity, and educational attainment with prediabetes and prediabetes awareness, controlling for sociodemographic, health and healthcare-related, and clinical characteristics.

**Results:**

In fully adjusted logistic regression models, Asian, Black, and Hispanic adults had a statistically significant higher risk of prediabetes than White adults (PR:1.26 [1.18,1.35], PR:1.17 [1.08,1.25], and PR:1.10 [1.02,1.19], respectively). Adults completing less than high school and high school had a significantly higher risk of prediabetes compared to those with a college degree (PR:1.14 [1.02,1.26] and PR:1.12 [1.01,1.23], respectively). We also found that Black and Hispanic adults had higher rates of prediabetes awareness in the fully adjusted model than White adults (PR:1.27 [1.07,1.50] and PR:1.33 [1.02,1.72], respectively). The rates of prediabetes awareness were consistently lower among those with less than a high school education relative to individuals who completed college (fully-adjusted model PR:0.66 [0.47,0.92]).

**Discussion:**

Disparities in prediabetes among racial and ethnic minority groups and adults with low educational attainment suggest challenges and opportunities for promoting health equity in high-risk groups and expanding awareness of prediabetes in the United States.

## 1 Introduction

U.S. adults from racial and ethnic minority groups, including non-Hispanic Asian, non-Hispanic Black, and Hispanic/Latino adults, experience a disproportionate burden of diabetes relative to non-Hispanic White adults. (1). These groups face higher diabetes prevalence, as well as higher rates of poor glycemic control, diabetes complications, and mortality (2). Diabetes disparities are also documented among adults with lower socio-economic status, especially those with low educational attainment, who exhibit higher diabetes prevalence and worse diabetes-related outcomes compared with their more educated counterparts (3–5).

Prediabetes represents a high-risk state for developing type 2 diabetes, characterized by blood glucose levels that are higher than normal but below diagnostic thresholds for diabetes (6). The American Diabetes Association defines prediabetes as a fasting plasma glucose level of 100 to 125 mg/dL, a 2-h plasma glucose after an oral glucose tolerance test of 140 to 199 mg/dL, or a glycated hemoglobin level of 5.7 to 6.4% (7). It is estimated that up to 50% of individuals with prediabetes progress to type 2 diabetes within 5 years, and 70% will develop the condition over their lifetime (6, 8). However, more than 80% of adults with prediabetes are unaware of their prediabetic status (9), hindering their ability to improve lifestyle behaviors or adopt evidence-based treatments that can lower diabetes risk (10).

Prior research examining the association of race and ethnicity or educational attainment with prediabetes prevalence and awareness have found no disparities in prediabetes. A recent analysis of nationally representative data found that age-adjusted prediabetes prevalence and awareness rates were similar across racial and ethnic groups and education levels (9). Another study of nationally representative survey data also reported no disparities in prediabetes prevalence by race or ethnicity (11). However, estimates of prediabetes prevalence and awareness in these studies were only adjusted for age, sex, and body mass index and not for other potential confounders such as household income, healthcare access, and other factors that are associated with these exposures and outcomes. Elucidating the multivariable association of race, ethnicity, and educational attainment with prediabetes could help inform strategies to support health equity in the prevention of diabetes and its complications (12–14).

To fill this evidence gap, our study had two aims: (1) Investigate the multivariable prevalence and prevalence ratio of prediabetes according to race, ethnicity, and educational attainment, and (2) Assess prediabetes awareness by race, ethnicity, and educational attainment among those with the condition.

## 2 Materials and methods

### 2.1 Study design and participants

This study represents a pooled cross-sectional analysis of National Health and Nutrition Examination Survey (NHANES) data spanning from 2011 to March 2020. NHANES is an ongoing stratified survey conducted by the Centers for Disease Control and Prevention (CDC)‘s National Center for Health Statistics (NCHS) (15) with data released every 2 years. The survey uses a complex, multistage, probability sampling design that makes findings representative of the U.S. civilian non-institutionalized population. Participants are interviewed at home and then invited to attend a mobile examination center to complete a health examination and laboratory measurements, including collection of blood specimens that include hemoglobin A1c (A1c). A subsample of participants is randomly selected to complete fasting blood collection, enabling measurement of fasting plasma glucose (FPG) levels. More information about the NHANES survey methods and laboratory measurements has been published previously (15, 16).

**Figure 1:**
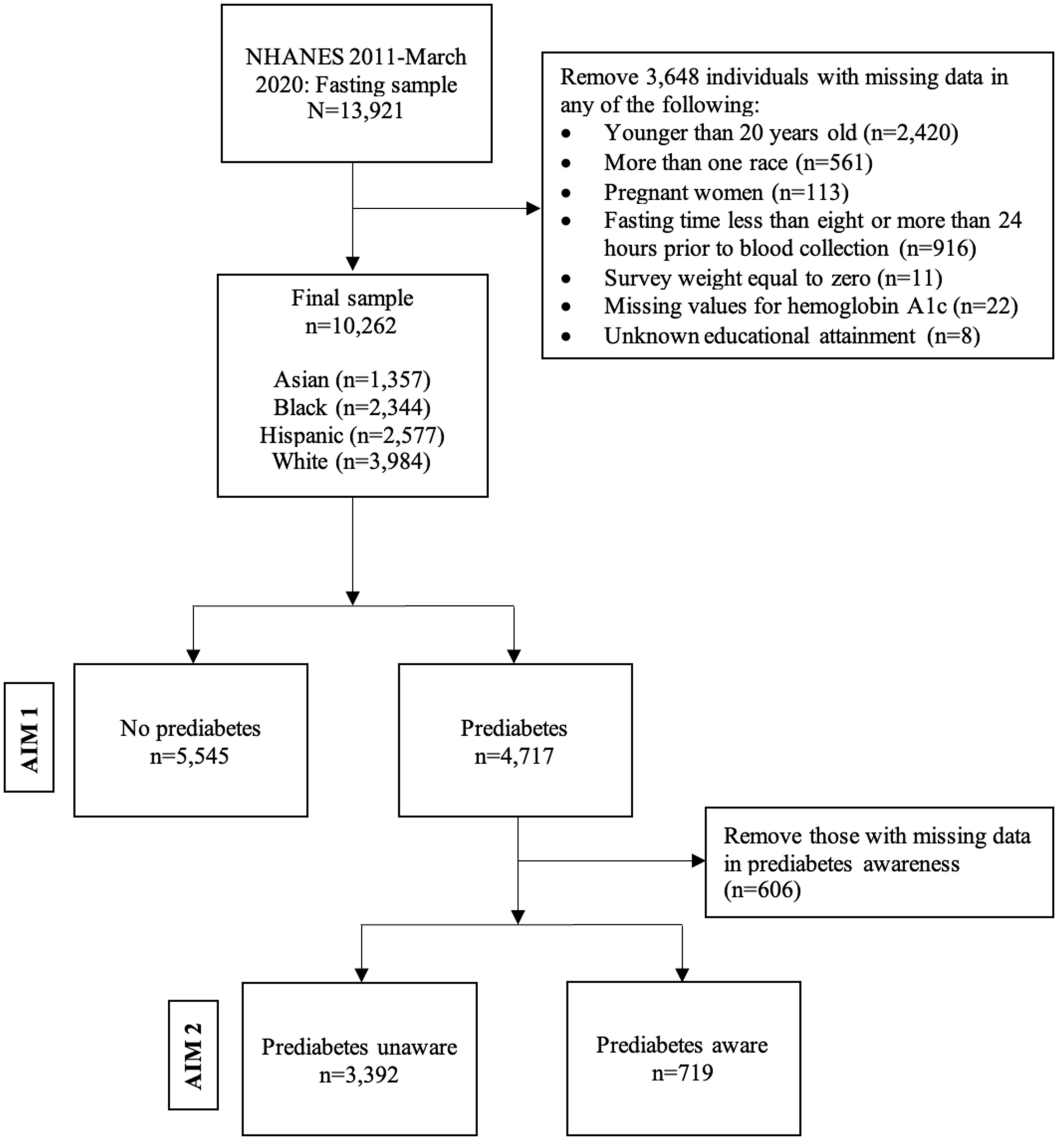
Flow chart of unweighted data, National Health and Nutrition Examination Survey 2011- March 2020, Fasting sample.

Overall, 45,462 individuals participated in the NHANES from 2011 to March 2020. Our initial sample included 13,921 individuals who were part of the fasting subsample with FPG data. The eligibility criteria for the current study were ages 20 years or older. Participants who reported “other race – including multi-racial” were excluded due to insufficient sample sizes that would preclude statistically reliable estimates. We also excluded pregnant women, those with less than 8 or more than 24 hours of fasting prior to blood collection, those with a survey weight equal to zero, individuals with missing values for A1c, and those with unknown educational attainment values. Our analytic sample for Aim 1 consisted of 10,262 adults that reported being non-Hispanic Asian, non-Hispanic Black, Hispanic/Latino, or non-Hispanic White. Aim 2 comprised 4,111 participants meeting glycemic criteria for prediabetes with information on prediabetes awareness (Figure 1).

### 2.2 Key variables

#### 2.2.1 Exposures

Major racial and ethnic groups sampled in NHANES include adults who reported being non-Hispanic Asian (i.e., those with origins in the Far East, Southeast Asia, or the Indian subcontinent), non-Hispanic Black, Hispanic, and non-Hispanic White adults. We classified individuals as non-Hispanic Asian (hereafter, Asian), non-Hispanic Black (hereafter, Black), Hispanic, or non-Hispanic White (hereafter, White).

Educational attainment was defined based on the question: “What is the highest grade or level of school you have completed or the highest degree you have received?” Participants’ responses were categorized into the following groups: “less than high school,” “high school,” “some college,” and “college or higher.” This categorization was based on common educational milestones reported in prior research (17, 18).

#### 2.2.2 Outcomes

Prediabetes was defined by A1c values of 5.7–6.4% or FPG of 100-125 mg/dL (19). Fasting glucose values were adjusted using backward regression equations provided by the NCHS to ensure comparability across sequential NHANES cycles (20). Self-reported prediabetes status was used to classify individuals as aware or unaware of prediabetes among those meeting glycemic criteria for prediabetes (*n* = 4,717).

Prediabetes awareness among those with prediabetes was defined by a positive response to the following question: “Have you ever been told by a doctor or other health professional that you have any of the following: prediabetes, impaired fasting glucose, impaired glucose tolerance, borderline diabetes, or that your blood sugar is higher than normal but not high enough to be called diabetes or sugar diabetes?” We also included those who reported being told by a doctor or other health professional they had “borderline” diabetes from the question: “Have you ever been told by a doctor or health professional that you have diabetes or sugar diabetes?”

#### 2.2.3 Covariates

We assessed sociodemographic, health and healthcare-related, and clinical variables as potential covariates. Sociodemographic variables included age, sex, and ratio of household family income to poverty. The latter variable was categorized based on the federal poverty level (FPL) (≤100% or > 100% FPL, respectively). Health and healthcare-related variables comprised performing any physical activity, current smoking status, alcohol use (weekly frequency), diet (recommended daily energy intake of ≤2,000 calories for women and ≤ 2,500 calories for men) (21), health insurance, and having a usual source of care. We also examined weight status categorized as body mass index (BMI) and waist circumference, given the association of obesity and abdominal obesity with elevated type 2 diabetes risk (22). BMI was calculated based on participants’ measured weight and height and was categorized according to the following widely accepted thresholds as underweight: ≤18.5 kg/m^2^; normal: 18.5–24.9 kg/m^2^; overweight: 25–29.9 kg/m^2^; and obesity: ≥30 kg/m^2^. According to commonly used cut-points, waist circumference was measured in centimeters and categorized by sex as normal (≤88 cm in women and ≤ 102 cm in men) or abdominal obesity (>88 cm in women and > 102 cm for men) among Black, Hispanic, and White adults. Waist circumference among Asian adults was categorized as normal (≤79 cm in women and ≤ 89 cm in men) or abdominal obesity (>79 cm in women and > 89 cm for men) (23).

### 2.3 Data analysis

All analyses used fasting sampling weights and accounted for the NHANES complex survey design (15). We used descriptive statistics to characterize the sample with respect to sociodemographic, health and healthcare-related, and clinical covariates. Categorical variables are presented by unweighted sample sizes and weighted percentages with their respective 95% confidence intervals (95% CI). Continuous variables are presented by weighted mean and standard errors (SE). These descriptive analyses were stratified by race, ethnicity, and educational attainment, the key independent variables. Chi-square and ANOVA tests were employed to examine bivariate relationships of the exposures with the covariates and the outcomes, namely prediabetes prevalence (Aim 1) and prediabetes awareness (Aim 2).

Logistic regression was used to model prediabetes prevalence (Aim 1) and prediabetes awareness (Aim 2) according to race, ethnicity, and educational attainment. We report the prevalence, prevalence ratios (PR), and 95% confidence intervals (CIs) for the two outcomes among Asian, Black, and Hispanic adults compared to White adults, and of those with less than a college degree (i.e., less than high school, a high school diploma or equivalent, or some college) compared to those who completed college. Unadjusted models (Model 1) were followed by three different models adjusting for successive groups of covariates to control for their potential confounding in the association between the exposure variables and prediabetes. Model 2 included age, gender, and the ratio of household family income to poverty (i.e., sociodemographic variables). Model 2 also included educational attainment in analyses examining racial and ethnic differences in the outcomes. Similarly, Model 2 included race and ethnicity for analyses examining differences in the outcomes by educational attainment. For Model 3, we additionally included physical activity, smoking status, alcohol use, daily energy intake, insurance, and usual source of care (i.e., health and healthcare-related characteristics). Model 4 included all prior covariates, in addition to weight status and waist circumference (i.e., clinical characteristics). A separate category for those missing data on the covariates was created to retain those individuals in the models. In addition, we tested the association between race and ethnicity and prediabetes or prediabetes awareness in analyses stratified by educational level. STATA SE software version 18 was used to carry out the analyses (24). SUDAAN 11 statistical package was used to calculate prevalence ratios (25). Statistical significance was determined based on *p*-values ≤0.05. NHANES data is publicly available online, and the coding scripts can be found on GitHub https://github.com/taynaraformagini/predmNHANES.

## 3 Results

### 3.1 Descriptive analysis

Table 1 displays the sociodemographic, health and healthcare-related, and clinical characteristics of the U.S. adult population by race and ethnicity. White adults were, on average, older than their Asian, Black, and Hispanic counterparts. Hispanic adults had lower educational attainment compared with the other groups. A higher percentage of Black and Hispanic adults exhibited incomes below the FPL in contrast to their Asian and White counterparts. Health insurance coverage was less prevalent among Black and Hispanic adults than among Asian and White adults, and fewer Hispanic adults had a usual source of care. The prevalence of obesity was highest among Black adults, followed by Hispanic, White, and Asian adults.

**Table 1:** Characteristics of U.S. adults 20 years or older by race and ethnicity, National Health and Nutrition Examination Survey 2011-March 2020, Fasting sample (*n* = 10,262).

	All	Asian^a^	Black^a^	Hispanic^a^	White^a^	*p*-value
	*n* (%)^b^	% (95% CI)^c^	% (95% CI)^c^	% (95% CI)^c^	% (95% CI)^c^	
Educational attainment^d^						
< High school	2,261 (15.6)	13.7 (11.3, 16.5)	16.3 (14.0, 18.9)	38.5 (35.0, 42.0)	10.4 (8.3, 13.0)	<0.001
High school	2,299 (21.8)	13.1 (10.6, 16.2)	27.5 (25.4, 29.7)	21.7 (19.3, 24.2)	21.5 (18.9, 24.4)	
Some college	3,070 (31.2)	19.1 (16.0, 22.5)	35.7 (32.8, 38.8)	25.6 (23.1, 28.1)	32.7 (30.4, 35.1)	
College	2,632 (31.4)	54.1 (49.4, 58.7)	20.5 (17.6, 23.7)	14.3 (11.7, 17.3)	35.4 (31.4, 39.5)	
Age, years						
Mean (SE)^e^	47.9 (0.3)	45.1 (0.6)	45.4 (0.4)	42.1 (0.5)	49.9 (0.4)	<0.001
Sex						
Female	5,273 (51.4)	53.1 (50.9, 55.2)	54.9 (52.6, 57.3)	49.9 (48.1, 51.7)	51.1 (49.5, 52.6)	<0.01
Federal poverty level (FPL)						
≤ 100%	2,027 (15.7)	13.4 (10.5, 16.8)	28.1 (23.9, 32.8)	33.6 (29.7, 37.8)	10.0 (8.0, 12.5)	<0.001
Physical activity						
Yes	7,661 (78.0)	76.5 (73.3, 79.3)	76.5 (74.2, 78.6)	74.9 (72.9, 76.7)	79.1 (77.2, 80.9)	0.001
Smoking status						
Yes	2,117 (22.2)	12.0 (9.7, 14.8)	33.6 (30.7, 36.6)	17.1 (15.3, 19.1)	22.1 (19.8, 24.7)	<0.001
Frequency of alcohol use						
No alcohol use	2,783 (24.9)	48.1 (44.1, 52.1)	30.4 (27.1, 34.0)	31.0 (28.3, 34.0)	20.9 (18.1, 23.9)	<0.001
≤ 2 days/week	4,786 (56.7)	43.1 (38.7, 47.6)	53.5 (50.7, 56.3)	60.7 (57.5, 63.7)	57.4 (54.7, 60.1)
> 2 days/week	1,282 (18.4)	8.8 (7.3, 10.7)	16.1 (13.5, 19.0)	8.3 (6.9, 10.0)	21.7 (19.5, 24.1)
Meets recommended daily energy intake (kcal)^f^						
Yes	6,304 (63.3)	74.9 (72.1, 77.4)	64.2 (60.9, 67.4)	61.3 (58.8, 63.7)	62.7 (60.5, 64.8)	<0.001
Health insurance						
Yes	8,281 (83.1)	85.6 (82.3, 88.4)	77.3 (74.1, 80.3)	61.1 (57.9, 64.2)	89.0 (87.1, 90.6)	<0.001
Usual source of care						
Yes	8,561 (83.9)	77.1 (73.8, 80.1)	84.3 (81.7, 86.5)	71.4 (69.0, 73.7)	87.2 (85.6, 88.7)	<0.001
Weight status						
Underweight	159 (1.6)	4.3 (3.1, 5.8)	1.6 (1.0, 2.3)	0.9 (0.5, 1.6)	1.5 (1.0, 2.0)	<0.001
Healthy weight	2,695 (27.1)	55.0 (52.2, 57.6)	21.3 (19.3, 23.5)	19.3 (17.2, 21.5)	27.6 (25.6, 29.7)	
Overweight	3,289 (33.0)	29.7 (27.0, 32.6)	29.3 (29.9, 31.7)	35.6 (33.3, 37.9)	33.2 (31.8, 34.6)	
Obesity	4,000 (38.4)	11.1 (9.3, 13.1)	47.8 (45.0, 50.7)	44.2 (41.6, 46.8)	37.8 (35.6, 40.0)	
Abdominal obesity						
Yes	5,592 (58.4)	62.2 (58.5, 65.7)^h^	60.1 (57.5, 63.0)	57.7 (55.0, 60.8)	60.3 (57.8, 62.8)	<0.001
Prediabetes prevalence						
Yes	4,717 (41.4)	39.4 (36.8, 42.0)	45.7 (42.9, 48.6)	41.6 (39.4, 43.8)	40.8 (38.6, 43.0)	<0.01
Prediabetes awareness^g^						
Yes	719 (15.4)	13.9 (10.4, 18.2)	17.0 (14.8, 19.5)	13.8 (11.4, 16.6)	15.5 (13.2, 18.2)	0.39

^a^: Asian = non-Hispanic Asian; Black = non-Hispanic Black; Hispanic = Hispanic or Latino; White = non-Hispanic White.

^b^: Unweighted sample size and weighted percentage of individuals. Where the total does not add to 10,262, participants were missing data on that characteristic. Weighted % is expressed as column percentages, which add to 100% across each characteristic.

^c^: The weighted % (95% CI) are expressed as column percentages, which add to 100% across each stratum.

^d^: < High school = less than high school; high school = completed high school; some college = some college but not graduate; college = college graduate or higher education.

^e^: Weighted mean; SE = standard error.

^f^: Recommended daily energy intake is ≤ 2,000 for women and ≤ 2,500 for men.

^g^: Prediabetes awareness was calculated among those with prediabetes and data on prediabetes awareness (*n* = 4,111).

^h^: Waist circumference cut-off for Asian adults was set at > 79 cm in women and > 89 cm for men.

We found the following unadjusted prevalence of prediabetes by race and ethnicity: Asian adults 39.4% (CI: 36.8, 42.0); White adults 40.8% (CI: 38.6, 43.0); Hispanic adults 41.6% (CI: 39.4, 43.8); and Black adults 45.7% (CI: 42.9, 48.6) (*p* < 0.01). Among those with prediabetes, awareness was low overall, with only 15.4% of adults meeting glycemic thresholds being aware of having the condition. There were no observed differences in prediabetes awareness by race or ethnicity (*p* = 0.39) (Table 1).

Table 2 describes the characteristics of the U.S. adult population by educational attainment. Adults who did not complete high school were much more likely to have household incomes below the FPL compared to those with higher educational levels. The proportion of adults with health insurance, a usual source of care, and practicing any physical activity was higher according to increasing levels of educational attainment. Obesity measured through BMI and abdominal obesity were lower among college graduates but similar among the other three educational attainment groups.

**Table 2:** Characteristics of U.S. adults 20 years or older by educational attainment, National Health and Nutrition Examination Survey 2011- March 2020, Fasting sample (*n* = 10,262).

	All	< High school^a^	High school^a^	Some college^a^	College^a^	*p*-value
	*n* (%)^b^	% (95% CI)^c^	% (95% CI)^c^	% (95% CI)^c^	% (95% CI)^c^	
Race and ethnicity^d^						
Asian	1,357 (5.7)	5.0 (3.7, 6.7)	3.4 (2.6, 4.5)	3.5 (2.9, 4.2)	9.8 (8.2, 11.7)	<0.001
Black	2,344 (11.8)	12.3 (98.3, 15.9)	14.8 (11.7, 18.6)	13.5 (11.1, 16.2)	7.7 (6.2, 9.4)	
Hispanic	2,577 (15.4)	38.0 (31.8, 44.4)	15.3 (12.4, 18.8)	12.6 (10.0, 15.7)	7.0 (5.5, 8.8)	
White	3,984 (67.1)	44.9 (37.3, 52.7)	66.4 (60.6, 71.8)	70.4 (65.8, 74.6)	75.5 (72.7, 78.1)	
Age, years						
Mean age (SE)^e^	47.9 (0.3)	49.5 (0.6)	48.9 (0.6)	46.3 (0.5)	48.0 (0.5)	<0.01
Sex						
Female	5,273 (51.4)	48.3 (45.6, 50.9)	49.1 (46.3, 51.9)	54.4 (52.3, 56.6)	51.7 (49.7, 53.6)	<0.01
Federal poverty level (FPL)						
≤ 100%	2,027 (15.7)	38.2 (34.1, 42.4)	19.8 (16.6, 23.5)	14.0 (12.2, 16.2)	4.4 (3.4, 5.6)	<0.001
Physical activity						
Yes	7,661 (78.0)	68.6 (65.4, 71.6)	75.6 (73.1, 77.9)	79.1 (76.7, 81.3)	83.3 (81.0, 85.3)	<0.001
Smoking status						
Yes	2,117 (22.2)	33.7 (29.6, 38.1)	31.4 (28.3, 34.7)	22.5 (20.3, 24.8)	9.9 (8.3, 11.8)	<0.001
Frequency of alcohol use						
No alcohol use	2,783 (24.9)	38.5 (35.0, 42.1)	28.3 (24.9, 31.9)	22.8 (20.1, 25.7)	18.9 (16.3, 21.8)	<0.001
≤ 2 days/week	4,786 (56.7)	49.6 (45.6, 53.6)	56.4 (53.0, 59.7)	60.3 (57.0, 63.4)	56.5 (54.6, 59.4)
> 2 days/week	1,282 (18.4)	11.9 (9.8, 14.4)	15.4 (12.8, 18.4)	17.0 (14.5, 19.8)	24.5 (21.5, 29,9)
Meets recommended daily energy intake (kcal)^f^						
Yes	6,304 (63.3)	66.3 (63.4, 69.1)	65.2 (62.4, 67.9)	63.1 (60.9, 65.1)	60.6 (57.7, 63.5)	0.02
Health insurance						
Yes	8,281 (83.1)	65.6 (61.6, 69.3)	78.7 (75.9, 81.2)	84.0 (81.8, 86.0)	94.1 (92.7, 95.3)	<0.001
Usual source of care						
Yes	8,561 (83.9)	76.4 (73.5, 79.0)	82.6 (80.2, 84.8)	85.1 (83.6, 86.6)	87.2 (84.6, 89.4)	<0.001
Weight status						
Underweight	159 (1.6)	1.8 (1.2, 2.7)	1.26 (0.8, 1.9)	1.36 (0.8, 2.1)	1.78 (1.1, 2.8)	<0.001
Healthy weight	2,695 (27.1)	25.3 (21.7, 29.3)	24.2 (21.4, 27.6)	23.7 (21.9, 25.7)	33.4 (30.2, 36.8)	
Overweight	3,289 (33.0)	32.1 (28.8, 35.6)	33.0 (29.8, 36.2)	32.6 (30.3, 34.9)	33.6 (31.5, 35.8)	
Obesity	4,000 (38.4)	40.8 (38.1, 43.5)	41.6 (38.5, 44.7)	42.4 (39.7, 45.0)	31.2 (28.2, 34.4)	
Abdominal obesity						
Yes	5,592 (58.4)	60.1 (57.8, 62.3)	61.7 (59.0, 64.3)	62.8 (59.6, 65.9)	50.9 (47.0, 54.8)	<0.001
Prediabetes prevalence						
Yes	4,717 (41.4)	49.3 (46.1, 52.5)	46.7 (43.9, 49.6)	38.3 (35.3, 41.4)	37.0 (34.2, 39.8)	<0.01
Prediabetes awareness^g^						
Yes	719 (15.4)	11.5 (9.5, 13.9)	15.4 (12.2, 19.3)	15.4 (12.7, 18.6)	17.7 (13.9, 22.2)	0.11

^a^: < High school = less than high school; high school = completed high school; some college = some college but not graduate; college = college graduate or higher education.

^b^: Unweighted sample size and weighted percentage of individuals. Where the total does not add to 10,262, participants were missing data on that characteristic. Weighted % is expressed as column percentages, which add to 100% across each characteristic.

^c^: The weighted % (95% CI) are expressed as column percentages, which add to 100% across each stratum.

^d^: Asian = non-Hispanic Asian; Black = non-Hispanic Black; Hispanic = Hispanic or Latino; White = non-Hispanic White.

^e^: Weighted mean; SE = standard error.

^f^: Recommended daily energy intake is ≤ 2,000 for women and ≤ 2,500 for men.

^g^: Prediabetes awareness was calculated among those with prediabetes and data on prediabetes awareness (*n* = 4,111).

Unadjusted analyses revealed an inverse relationship between educational attainment and prediabetes prevalence. A total of 49.3% (CI: 46.1, 52.5) of adults with less than high school education and 46.7% (CI: 43.9, 49.6) of adults who completed only high school had prediabetes, compared with 38.3% (CI: 35.3, 41.4) of those with some college and 37.0% (CI: 34.2, 39.8) of those with a college degree. Awareness of prediabetes was lower among adults who did not complete high school (11.5%) compared to those with a college degree (17.7%), but this difference was not statistically significant (*p* = 0.11) (Table 2).

### 3.2 Aim 1: prediabetes

We assessed the prevalence and prevalence ratios of prediabetes among groups defined by race, ethnicity, and educational attainment (Table 3). In unadjusted analyses, Black adults had a higher prevalence of prediabetes compared to their White counterparts (PR:1.12 [1.04, 1.21]). When adjusting for sociodemographic characteristics, we found that Asian, Black, and Hispanic adults had a statistically significantly higher prevalence of prediabetes than White adults (PR:1.10 [1.02, 1.17], PR:1.21 [1.13, 1.29], and PR:1.14 [1.05, 1.23], respectively). This statistical significance was maintained when adjusting for health and healthcare-related variables and clinical variables (PR:1.26 [1.18, 1.35], PR:1.17 [1.08, 1.25], and PR:1.10 [1.02, 1.19], respectively).

**Table 3:** Prevalence of prediabetes by race, ethnicity, and educational attainment among U.S. adults, National Health and Nutrition Examination Survey 2011-March 2020, Fasting Sample (*n* = 10,262).

Prediabetes	Model 1^b^		Model 2^c^		Model 3^d^		Model 4^e^	
	Prevalence (95% CI)	PR (95% CI)	Prevalence (95% CI)	PR (95% CI)	Prevalence (95% CI)	PR (95% CI)	Prevalence (95% CI)	PR (95% CI)
Race and ethnicity^a^								
Asian	39.4 (36.9, 42.0)	0.97 (0.90, 1.04)	43.2 (40.7, 45.7)	1.10 (1.02, 1.17)*	44.0 (41.2, 46.7)	1.11 (1.04, 1.19)*	49.7 (46.9, 52.6)	1.26 (1.18, 1.35)*
Black	45.7 (43.0, 48.6)	1.12 (1.04, 1.21)*	47.7 (45.1, 50.2)	1.21 (1.13, 1.29)*	47.1 (44.6, 49.7)	1.19 (1.11, 1.28)*	46.0 (43.4, 48.7)	1.17 (1.08, 1.25)*
Hispanic	41.6 (39.4, 43.8)	1.02 (0.95, 1.09)	44.9 (42.3, 47.5)	1.14 (1.05, 1.23)*	44.7 (42.4, 47.3)	1.13 (1.05, 1.22)*	43.6 (41.2, 45.9)	1.10 (1.02, 1.19)*
White	40.8 (38.6, 43.0)	1 [Reference]	39.4 (37.2, 41.7)	1 [Reference]	39.5 (37.3, 41.7)	1 [Reference]	39.5 (37.3, 41.7)	1 [Reference]
Educational attainment^f^								
< High school	49.3 (46.1, 52.5)	1.33 (1.20, 1.48)*	45.8 (42.3, 49.3)	1.21 (1.09, 1.34)*	45.2 (41.7, 48.7)	1.17 (1.05, 1.31)*	45.0 (41.5, 48.6)	1.14 (1.02, 1.26)*
High school	46.7 (43.9, 49.6)	1.26 (1.14, 1.40)*	45.3 (42.7, 47.9)	1.20 (1.09, 1.32)*	44.7 (42.1, 47.4)	1.16 (1.06, 1.28)*	44.3 (41.6, 46.9)	1.12 (1.02, 1.23)*
Some college	38.3 (35.3, 41.4)	1.04 (0.94, 1.14)	40.1 (37.2, 43.0)	1.06 (0.97, 1.16)	40.1 (37.3, 43.0)	1.04 (0.94, 1.14)	39.4 (36.6, 42.2)	1.00 (0.91, 1.08)
College	37.0 (34.2, 39.8)	1 [Reference]	37.9 (35.3, 40.5)	1 [Reference]	38.5 (36.0, 41.1)	1 [Reference]	39.6 (37.1, 42.1)	1 [Reference]

CI, confidence interval; PR, prevalence ratio.

^a^: Asian = non-Hispanic Asian; Black = non-Hispanic Black; Hispanic = Hispanic or Latino; White = non-Hispanic White.

^b^: unadjusted.

^c^: adjusted for socio-demographic variables (age, gender, and family income) + educational attainment for the race and ethnicity regressions and race and ethnicity for the educational attainment regressions.

^d^: adjusted for socio-demographic variables and health and healthcare-related variables (physical activity, smoking status, alcohol use, recommended daily energy intake, insurance, and usual source of care).

^e^: adjusted for socio-demographic variables, health and healthcare-related variables, and clinical variables (weight status and waist circumference).

^f^: < High school = less than high school; high school = completed high school; some college = some college but not graduate; college = college graduate or higher education.

*: statistically significant at *p*-value ≤ 0.05.

In unadjusted analyses, adults with less than high school and high school completion had a significantly higher prevalence of prediabetes (PR:1.33 [1.20, 1.48] and PR:1.26 [1.14, 1.40], respectively) compared to those with a college degree. This association was attenuated although still statistically significant in all three subsequent models adjusting for sociodemographic, health and healthcare-related, and obesity-related variables. In the fully adjusted model, adults completing less than high school and high school had a significantly higher prevalence of prediabetes compared to adults with a college degree (PR:1.14 [1.02, 1.26] and PR:1.12 [1.02, 1.23], respectively). There were no significant differences in prediabetes between those who completed some college vs. those with a college degree.

### 3.3 Aim 2: prediabetes awareness

Table 4 presents the prevalence and prevalence ratios of prediabetes awareness among those with prediabetes by race, ethnicity, and educational attainment. For race and ethnicity, Black adults exhibited higher awareness of prediabetes when adjusting for sociodemographic variables (PR: 1.28 [1.07, 1.53]). The fully adjusted prevalence ratio of Black and Hispanic adults demonstrated that these two groups had higher awareness of prediabetes than White adults (PR:1.27 [1.07, 1.50] and PR:1.33 [1.02, 1.72], respectively).

**Table 4:** Prevalence of prediabetes awareness among those with prediabetes by race, ethnicity, and educational attainment among U.S. adults, National Health and Nutrition Examination Survey 2011-March 2020, Fasting Sample (*n* = 4,111).

Prediabetes awareness	Model 1^b^		Model 2^c^		Model 3^d^		Model 4^e^		Prevalence (95% CI)	PR (95% CI)	Prevalence (95% CI)	PR (95% CI)	Prevalence (95% CI)	PR (95% CI)	Prevalence (95% CI)	PR (95% CI)
Race and ethnicity ^a^								
Asian	13.9 (10.5, 18.2)	0.89 (0.66, 1.21)	14.2 (10.7, 18.7)	0.99 (0.73, 1.34)	14.0 (10.5, 18.4)	0.97 (0.71, 1.33)	17.7 (13.5, 22.9)	1.25 (0.93, 1.68)
Black	17.0 (14.8, 19.6)	1.10 (0.91, 1.32)	18.5 (16.2, 21.0)	1.28 (1.07, 1.53)*	18.3 (15.9, 20.9)	1.27 (1.06, 1.52)*	18.0 (15.6, 20.6)	1.27 (1.07 1.50)*
Hispanic	13.8 (11.4, 16.6)	0.89 (0.68, 1.16)	18.2 (15.0, 21.9)	1.26 (0.97, 1.63)	18.8 (15.4, 22.8)	1.30 (1.00, 1.70)*	18.8 (15.4, 22.8)	1.33 (1.02, 1.72)*
White	15.5 (13.2, 18.2)	1 [Reference]	14.4 (12.3, 16.9)	1 [Reference]	14.4 (12.3, 16.9)	1 [Reference]	14.2 (12.1, 16.6)	1 [Reference]
Educational attainment ^f^								
< High school	11.5 (9.5, 13.9)	0.65 (0.48, 0.88)*	11.5 (9.3, 14.3)	0.64 (0.47, 0.88)*	11.8 (9.3, 14.8)	0.67 (0.48, 0.94)*	11.9 (9.4, 14.9)	0.66 (0.47, 0.92)*
High school	15.4 (12.2, 19.3)	0.87 (0.61, 1.24)	15.1 (11.9, 19.1)	0.85 (0.59, 1.21)	15.1 (11.9, 19.0)	0.85 (0.60, 1.22)	15.1 (11.9, 18.9)	0.84 (0.58, 1.20)
Some college	15.4 (12.7, 18.6)	0.87 (0.67, 1.13)	15.5 (12.7, 18.7)	0.86 (0.66, 1.14)	15.4 (12.7, 18.6)	0.87 (0.65, 1.15)	15.1 (12.5, 18.1)	0.84 (0.64, 1.10)
College	17.7 (13.9, 22.2)	1 [Reference]	17.9 (14.1, 22.4)	1 [Reference]	17.7 (13.9, 22.3)	1 [Reference]	18.0 (14.2, 22.6)	1 [Reference]

CI, confidence interval; PR, prevalence ratio.

^a^: Asian = non-Hispanic Asian; Black = non-Hispanic Black; Hispanic = Hispanic or Latino; White = non-Hispanic White.

^b^: unadjusted.

^c^: adjusted for socio-demographic variables (age, gender, and family income) + educational attainment for the race and ethnicity regressions and race and ethnicity for the educational attainment regressions.

^d^: adjusted for socio-demographic variables and health and healthcare-related variables (physical activity, smoking status, alcohol use, recommended daily energy intake, insurance, and usual source of care).

^e^: adjusted for socio-demographic variables, health and healthcare-related variables, and clinical variables (weight status and waist circumference).

^f^: < High school = less than high school; high school = completed high school; some college = some college but not graduate; college = college graduate or higher education.

*: statistically significant at *p*-value ≤ 0.05.

Prediabetes awareness estimates were consistently lower among those with less than a high school education relative to individuals who completed college. In the fully adjusted model, the proportion of adults with prediabetes who completed less than high school and were aware of their condition was 34% (0.47, 0.92) lower compared to those with a college degree.

### 3.4 Prediabetes stratified by educational attainment

The association between race and ethnicity and the prevalence of prediabetes stratified by educational category was also assessed (Supplementary Table S1). Fully adjusted models (Model 4) revealed that racial and ethnic minorities tended to have a higher prevalence of prediabetes compared to White adults when stratified by education. Statistically significant higher rates of prediabetes were found for Asian and Black adults with high school education, Asian, Black, and Hispanic adults with some college, and Black adults who were college graduates. We found no statistically significant associations between race and ethnicity and the prevalence of prediabetes awareness when stratified by educational attainment (Supplementary Table S2).

## 4 Discussion

To our knowledge, this is the first study examining racial, ethnic, and educational disparities in prediabetes prevalence and awareness that has adjusted for a broad set of covariates related to these exposures and outcomes. Our analyses of a large, nationally representative sample of U.S. adults found significant racial and ethnic disparities in prediabetes after adjusting for sociodemographic, health and healthcare-related, and clinical characteristics. Our findings also suggest an educational gradient in prediabetes, revealing an inverse relationship between educational attainment and prediabetes prevalence. Awareness of prediabetes was low in the U.S. adult population overall, estimated at 15%, and in all racial and ethnic and educational groups, ranging from 12% among those not completing high school to 18% among those with a college education. Multivariable analyses revealed a higher rate of prediabetes awareness among Black and Hispanic adults than White adults. We found lower rates of prediabetes awareness among those with less formal education. However, the difference in this outcome was only significant between those who had less than a high school education vs. college graduates. These findings have implications for achieving health equity in preventing diabetes and its complications.

In contrast to a large body of epidemiologic research that has consistently documented racial and ethnic disparities in type 2 diabetes and obesity (1, 26, 27), prior studies examining prediabetes prevalence by race and ethnicity have not found significant disparities (9, 11). These earlier analyses of prediabetes prevalence using nationally representative data adjusted only for age, sex, and/or body mass index. Our study, which also analyzed NHANES data, found racial/ethnic disparities in prediabetes by including 10 years of data and adjusting for a broader set of characteristics that are potential confounders given their association with race and ethnicity and cardiometabolic outcomes. There is scientific consensus that race and ethnicity represent social constructs rather than biological ones (28). Therefore, racial and ethnic disparities in prediabetes prevalence observed in this analysis likely reflect variations in unmeasured social factors associated with race and ethnicity and diabetes risk. These include psychological stress and trauma, neighborhood resources for access to healthy food and physical activity, cultural differences, and other environmental exposures (1, 29, 30).

There is little prior research on the relationship between educational attainment and prediabetes prevalence in the U.S. Recent national estimates based on data spanning from 2017 to 2020 found a similar age-adjusted prevalence of prediabetes across educational levels (9). Our multivariable analysis of data from 2011 to 2020 found that the risk of prediabetes was greater among U.S. adults with either high school or less formal education than those who completed college. This finding is supported by international studies documenting a similar inverse relationship between prediabetes prevalence and educational attainment in several Middle Eastern and Asian countries (31–34). A large body of literature in the U.S. and other high-income countries has established an inverse association between educational background and diabetes prevalence (3, 35–37). Among adults with diabetes, research has also demonstrated an educational gradient in glycemic control, diabetic complications, and mortality (38–41). Therefore, the inverse association we observed between educational attainment and prediabetes prevalence is consistent with many similar studies, yet further research on this topic in U.S. populations is needed. Future research could also examine more granular indicators of educational background than years of schooling, including skills learned or background knowledge that relates to healthy lifestyle change for preventing type 2 diabetes.

Awareness of prediabetes is low among U.S. adults, estimated at 15% of those with the condition. In prior research, there has been little focus on differences in prediabetes awareness by educational background. The most recent estimates from nationally representative data indicate comparable awareness of prediabetes across the following categories of educational attainment: less than high school (16.3%), high school completion (16.3%), and more than high school (18.3%) (9). Earlier analyses of the NHANES report conflicting findings on the relationship between prediabetes awareness and educational attainment, with some studies showing significant differences across educational groups and others not (42–44). All of these previous analyses were either unadjusted or adjusted for sex alone. We found educational disparities in prediabetes awareness among those with less than a high school education compared with college graduates, even after controlling for a broad array of potential confounders. This finding is consistent with many studies reporting educational gradients in health-related knowledge and health outcomes (45, 46). Educational disparities in these areas are likely related to the fact that those with lower education are less likely to have health insurance and a usual source of care (1). These findings might also be related to unmeasured social factors that are associated with formal education and confer health benefits (47–49). More research is needed to elucidate the mechanisms by which educational attainment impacts prediabetes awareness and develop interventions that can promote prediabetes awareness among those with less formal education.

Few prior studies examining prediabetes awareness have reported on differences by race and ethnicity. Earlier analyses of nationally representative data reported unadjusted or age-adjusted estimates of prediabetes awareness that did not differ significantly by race or ethnicity (9, 43, 44). Our fully adjusted model found that Black and Hispanic adults with prediabetes exhibited higher rates of prediabetes awareness than White individuals. In addition, prediabetes awareness was also higher among Asian adults compared to White adults in our fully adjusted model, although the estimates did not achieve statistical significance. The discrepancy between our findings and those reported previously likely stems from our adjustment for covariates that are related to race and ethnicity and prediabetes awareness. Higher prediabetes awareness among Black and Hispanic individuals may relate to the increased diabetes risk observed in these groups (26, 50, 51), which may heighten levels of awareness as healthcare providers might be more likely to test individuals with higher risk factors. Higher awareness of prediabetes in these groups may also reflect a greater likelihood of having family members with diabetes, which is twice as likely among Hispanic and Black adults than their White counterparts (52).

Our findings describing racial and ethnic and educational disparities in prediabetes prevalence and awareness may have implications for health equity. The higher prevalence of prediabetes, as well as many related cardiometabolic conditions (27, 53–55), observed among racial and ethnic minority groups and those with low educational attainment highlights the need to address diverse social determinants that raise disease risk in these groups (48, 56). Many of these factors, such as limited access to healthy foods and safe areas for physical activity, may be improved most effectively through changes in policy and community development. Prior research has also reported fewer evidence-based type 2 diabetes prevention programs located in low-income communities (57, 58), in addition to lower levels of participation in these programs among racial and ethnic minority groups and adults with less formal education (59). Given the potential for diabetes prevention programs to reduce type 2 diabetes incidence by up to 58% (60), increasing access in communities with limited educational resources and high proportions of racial and ethnic minority groups is recommended to be an urgent priority for promoting health equity.

Our examination of prediabetes awareness revealed that adults with less formal education had lower rates of being aware of prediabetes. Prior studies using diverse data sources and analytic methods have reported conflicting findings about whether awareness of prediabetes impacts physical activity or dietary behaviors known to help prevent type 2 diabetes (10, 42, 43, 61, 62). Even if prediabetes awareness is not associated with self-guided healthy lifestyle changes, individuals who know they have prediabetes at least have the opportunity to join guided, group-based type 2 diabetes prevention programs. Those unaware of prediabetes would not pursue this evidence-based treatment option that has the greatest potential to lower their diabetes risk.

This study’s finding that Black and Hispanic adults have higher prediabetes awareness than White adults in the fully adjusted model represents an opportunity to promote health equity in diabetes prevention. For these high-risk groups that experience a disproportionate burden of diabetes, being aware of prediabetes represents the first step toward making healthy lifestyle changes or adopting evidence-based treatment. The increase in prediabetes awareness among all U.S. adults over the last 15 years also suggests potential for population-based type 2 diabetes prevention efforts (63). However, with only 15% of U.S. adults with prediabetes being aware of their condition, much work is needed by public health practitioners and clinicians to assess diabetes risk and communicate the findings effectively to affected individuals and communities.

To our knowledge, this is the first study examining the multivariable association of race, ethnicity, and educational attainment with prediabetes outcomes. This is especially important given many potential confounders measured in NHANES that are associated with our exposures and outcomes, but have not yet been analyzed in prior research on this topic. The current study contributes new data to the field that can inform efforts to ensure health equity in type 2 diabetes prevention. Other strengths of this study include analyzing data that are representative of the entire U.S. adult population. Glycemic measurements in NHANES enable complete capture of prediabetes diagnoses based on abnormal results. In addition, we included the latest NHANES data to capture observed changes in prediabetes prevalence and awareness over the last decade.

Our study also has notable limitations. Cross-sectional studies like NHANES are not designed to enable causal inferences about the relationship of race, ethnicity, and educational attainment with prediabetes outcomes. We categorized prediabetes solely based on A1c and FPG because the 2017-March 2022 NHANES dataset does not include the 2-h oral glucose tolerance test (OGTT). As a result, individuals exhibiting positive OGTT results but negative A1c and FPG values were excluded from the analysis. NHANES participants in our youngest age category (i.e., 20–25 years old) may not have had the opportunity to complete college and may, therefore, have been classified with a lower level of educational attainment than they will eventually achieve based on their age. Some participants may have received the diagnosis of prediabetes from a healthcare provider but did not recall the event during NHANES interviews. It is also possible that some participants included in our analysis might have been diagnosed with prediabetes by a healthcare provider and were never informed of their condition. Infrequent documentation of prediabetes by primary care providers suggests that this may be a common occurrence (64). Finally, although we controlled for potential confounders associated with the exposures and outcomes, there may be residual confounding by unmeasured factors.

### 4.1 Conclusion

In a large nationally representative sample of U.S. adults, prediabetes prevalence was high, and awareness was low. Disparities in prediabetes prevalence and awareness among racial and ethnic minority groups and adults with low educational attainment suggest challenges and opportunities for promoting health equity in these high-risk groups. Increasing access to evidence-based type 2 diabetes prevention programs for all Americans will likely require targeted efforts in communities with high proportions of racial and ethnic minority groups and residents with low educational attainment, where these programs are less widely available (57). More research is needed to develop and test interventions promoting awareness of prediabetes. Motivational interventions promoting prediabetes awareness in clinical or community settings may produce greater uptake of healthy lifestyle behaviors and structured type 2 diabetes prevention programs than brief encounters with healthcare providers, where prediabetes diagnoses are most commonly communicated. Addressing unmeasured social factors that underlie our study findings, such as poor availability of healthy foods and other neighborhood-level exposures, may also promote health equity in type 2 diabetes prevention. Policy and community development interventions could be needed to impact these upstream factors, and future research is needed to study the effectiveness of these efforts.

## Data availability statement

The datasets presented in this study can be found in online repositories. The names of the repository/repositories and accession number(s) can be found at: https://github.com/taynaraformagini/predmNHANES.

## Ethics statement

This study uses non-identifiable data from the National Health and Nutrition Examination Survey (NHANES), a publicly available U.S. national dataset. The NHANES is approved by the National Center for Health Statistics (NCHS) Ethics Review Board. The NHANES is conducted in accordance with national legislation and institutional requirements. The human samples used in this study are non-identifiable and acquired from NHANES. NHANES obtained written informed consent from participants and so written informed consent was not required for this study.

## Author contributions

TF: Conceptualization, Data curation, Formal analysis, Investigation, Methodology, Project administration, Software, Writing – original draft, Writing – review & editing. JB: Conceptualization, Methodology, Supervision, Writing – original draft, Writing – review & editing. AR: Conceptualization, Data curation, Methodology, Software, Supervision, Writing – review & editing. KB: Conceptualization, Data curation, Methodology, Supervision, Writing – review & editing. YZ: Conceptualization, Methodology, Writing – review & editing. RS: Data curation, Formal analysis, Methodology, Software, Writing – review & editing. MJO: Conceptualization, Data curation, Formal analysis, Investigation, Methodology, Supervision, Writing – original draft, Writing – review & editing.
