# Supplementary material for: Prediabetes prevalence and awareness by race, ethnicity, and educational attainment among U.S. adults
Source: Front Public Health. 2023 Dec 18;11:1277657. doi: 10.3389/fpubh.2023.1277657 (PMC10758124; doi:10.3389/fpubh.2023.1277657)
Supplement: Supplementary file 1 [file Table_1.docx]

Supplementary Material

**Prediabetes Prevalence and Awareness by Race, Ethnicity, and Educational Attainment among U.S. Adults**

**Taynara Formagini ^1,2*^, Joanna Veazey Brooks ^2,3,4^, Andrew Roberts ^2,5^, Kai McKeever Bullard ^6^, Yan Zhang ^6^, Ryan Saelee ^6^, Matthew James O’Brien ^7^**

***** **Correspondence:**

Corresponding author

Taynara Formagini

9500 Gilman Dr. La Jolla, CA 92093

[tformgini@health.ucsd.edu](mailto:tformgini@health.ucsd.edu)

# Supplementary Data

*1.2 Prediabetes and prediabetes awareness by race and ethnicity stratified by educational attainment*

We tested the effect of race and ethnicity on the prevalence and prevalence ratio of prediabetes and prediabetes awareness using logistic regression models in stratified analyses by each educational category. Table 1 demonstrates the prevalence of prediabetes by race and ethnicity. Fully adjusted models (Model 4) revealed that racial and ethnic minorities tended to have higher prediabetes rates compared to White adults when stratified by education. Statistically significant higher rates of prediabetes were found for Asian and Black adults with high school education, Asian, Black, and Hispanic adults with some college, and Black adults who were college graduates. We found non-statistically significant effects for prediabetes awareness among Asian, Black, and Hispanic adults compared to White adults when stratified by educational attainment (Table 2).

**Table 1. Prevalence of prediabetes by race and ethnicity among U.S. adults stratified by educational attainment, National Health and Nutrition Examination Survey 2011-March 2020, Fasting Sample**

|  | **< High school** ^a^ | | **High school** ^a^ | | **Some college** ^a^ | | **College** ^a^ | |
| --- | --- | --- | --- | --- | --- | --- | --- | --- |
| **Race and ethnicity ^d^** | Model 4 ^c^  n=2,261 | | Model 4 ^c^  n=2,299 | | Model 4 ^c^  n=3,070 | | Model 4 ^c^  n=2,632 | |
|  | Prevalence  (95% CI) | PR  (95% CI) | Prevalence  (95% CI) | PR  (95% CI) | Prevalence  (95% CI) | PR  (95% CI) | Prevalence  (95% CI) | PR  (95% CI) |
| Asian | 56.5 (47.0, 65.5) | 1.19 (0.94, 1.52) | 55.4 (47.4, 63.1) | 1.26 (1.07, 1.47)* | 48.7 (40.9, 56.5) | 1.35 (1.10, 1.64)* | 39.7 (34.6, 45.1) | 1.11 (0.99, 1.26) |
| Black | 50.7 (45.3, 56.1) | 1.07 (0.88, 1.30) | 53.0 (47.6, 58.3) | 1.20 (1.05, 1.38)* | 43.4 (40.0, 46.8) | 1.20 (1.06, 1.36)* | 45.8 (39.7, 52.1) | 1.29 (1.10, 1.50)* |
| Hispanic | 50.2 (46.7, 53.6) | 1.06 (0.90, 1.26) | 50.1 (44.9, 55.3) | 1.14 (0.97, 1.33) | 42.5 (38.1, 47.1) | 1.18 (1.02, 1.36)* | 37.7 (32.4, 43.4) | 1.06 (0.90, 1.25) |
| White | 47.3 (40.3, 54.4) | 1 [Reference] | 44.1 (40.0, 48.2) | 1 [Reference] | 36.2 (32.3, 40.3) | 1 [Reference] | 35.7 (32.7, 38.8) | 1 [Reference] |

a: < High school = less than high school; high school= completed high school; some college=some college but not graduate; college=college graduate or higher education.

b: Asian= non-Hispanic Asian; Black= non-Hispanic Black; Hispanic= Hispanic or Latino; White= non-Hispanic White.

c: adjusted for age, gender, family income, physical activity, smoking status, alcohol use, recommended daily energy intake, insurance, usual source of care, weight status, and waist circumference.

*: statistically significant at p-value ≤ 0.05

**Table 2. Prevalence of prediabetes awareness among U.S. adults with prediabetes by race and ethnicity stratified by educational attainment, National Health and Nutrition Examination Survey 2011-March 2020, Fasting Sample**

|  | **< High school** ^a^ | | **High school** ^a^ | | **Some college** ^a^ | | **College** ^a^ | |
| --- | --- | --- | --- | --- | --- | --- | --- | --- |
| **Race and ethnicity ^d^** | Model 4 ^c^  n=2,261 | | Model 4 ^c^  n=2,299 | | Model 4 ^c^  n=3,070 | | Model 4 ^c^  n=2,632 | |
|  | Prevalence  (95% CI) | PR  (95% CI) | Prevalence  (95% CI) | PR  (95% CI) | Prevalence  (95% CI) | PR  (95% CI) | Prevalence  (95% CI) | PR  (95% CI) |
| Asian | - | - | - | - | 18.3 (10.0, 31.1) | 1.26 (0.69, 2.29) | 16.8 (11.7, 23.4) | 1.00 (0.63, 1.58) |
| Black | 16.0 (11.1, 22.5) | 1.72 (0.97, 3.05) | 12.3 (8.6, 17.5) | 0.81 (0.52, 1.26) | 20.2 (15.2, 26.4) | 1.39 (0.99, 1.95) | 23.0 (17.6, 29.5) | 1.37 (0.94, 1.98) |
| Hispanic | 14.8 (11.1, 19.5) | 1.60 (0.94, 2.71) | 20.5 (13.7, 29.5) | 1.34 (0.77, 2.34) | 16.1 (11.4, 22.2) | 1.10 (0.72, 1.69) | 25.1 (15.1, 38.6) | 1.49 (0.81, 2.75) |
| White | 9.3 (6.0, 14.1) | 1 [Reference] | 15.3 (11.0, 20.8) | 1 [Reference] | 14.6 (11.3, 18.6) | 1 [Reference] | 16.8 (12.3, 22.6) | 1 [Reference] |

a: < High school = less than high school; high school= completed high school; some college=some college but not graduate; college=college graduate or higher education.

b: Asian= non-Hispanic Asian; Black= non-Hispanic Black; Hispanic= Hispanic or Latino; White= non-Hispanic White.

c: adjusted for age, gender, family income, physical activity, smoking status, alcohol use, recommended daily energy intake, insurance, usual source of care, weight status, and waist circumference.

*: statistically significant at p-value ≤ 0.05

Some values were suppressed since the relative standard error was>30%, according to the National Center for Health Statistics recommendation.
